# Direct and indirect links between children’s socio-economic status and education: pathways via mental health, attitude, and cognition

**DOI:** 10.1007/s12144-021-02232-2

**Published:** 2021-09-04

**Authors:** Edwin S. Dalmaijer, Sophie G. Gibbons, Giacomo Bignardi, Alexander L. Anwyl-Irvine, Roma Siugzdaite, Tess A. Smith, Stepheni Uh, Amy Johnson, Duncan E. Astle

**Affiliations:** MRC Cognition and Brain Sciences Unit, University of Cambridge, 15 Chaucer Road, Cambridge, CB2 7EF UK

**Keywords:** Child development, Socioeconomic status, Network analysis, Cognitive ability, Reading, Maths, Anxiety, Depression, Grit, Growth mindset, Poverty

## Abstract

A child’s socio-economic environment can profoundly affect their development. While existing literature focusses on simplified metrics and pair-wise relations between few variables, we aimed to capture complex interrelationships between several relevant domains using a broad assessment of 519 children aged 7–9 years. Our analyses comprised three multivariate techniques that complimented each other, and worked at different levels of granularity. First, an exploratory factor analysis (principal component analysis followed by varimax rotation) revealed that our sample varied along continuous dimensions of cognition, attitude and mental health (from parallel analysis); with potentially emerging dimensions speed and socio-economic status (passed Kaiser’s criterion). Second, k-means cluster analysis showed that children did not group into discrete phenotypes. Third, a network analysis on the basis of bootstrapped partial correlations (confirmed by both cross-validated LASSO and multiple comparisons correction of binarised connection probabilities) uncovered how our developmental measures interconnected: educational outcomes (reading and maths fluency) were directly related to cognition (short-term memory, number sense, processing speed, inhibition). By contrast, mental health (anxiety and depression symptoms) and attitudes (conscientiousness, grit, growth mindset) showed indirect relationships with educational outcomes via cognition. Finally, socio-economic factors (neighbourhood deprivation, family affluence) related directly to educational outcomes, cognition, mental health, and even grit. In sum, cognition is a central cog through which mental health and attitude relate to educational outcomes. However, through direct relations with all components of developmental outcomes, socio-economic status acts as a great ‘unequaliser’.

**Supplementary Information:**

The online version contains supplementary material available at 10.1007/s12144-021-02232-2.

## Introduction

Growing up in relative poverty can profoundly affect children’s educational attainment. Children from poorer backgrounds typically start school at a disadvantage relative to their peers, and this gap widens over the school years (Andrews et al., 2017). This relationship is not merely correlational: even small increases in income can improve disadvantaged children’s school readiness (Dearing et al., 2001). The impacts of early socio-economic disadvantage can last a lifetime. It is associated with a poorer transition into the labour market (Gregg & Machin, 2001), and higher odds for low income, low qualifications, worklessness, and depression (Feinstein & Bynner, 2004). This long-term impact can create an intergenerational cycle of disadvantage, with risk factors cascading to future generations (Griggs & Walker, 2008; N. Smith & Middleton, 2007). Redressing the attainment gap is a major priority for policymakers (Andrews et al., 2017; HM Treasury, 2008) who seek to reduce educational underattainment, boost economic prospects, reduce the burden of chronic physical illness, and tackle the burgeoning mental health crisis in children and adolescents (Gunnell et al., 2018; McMillan et al., 2017).

While income is important, it is not the sole driver of socio-economic disadvantage. Socio-economic status (SES) is a multi-faceted construct, with many potentially active ingredients within children’s home life and environment (Bateman, 2014). SES-components that are directly related to income are deprivation of basic (food, clothing, bills) and secondary lifestyle (car, microwave, dishwasher), housing facilities (shower, flushing toilet, hot water) and deterioration (leaks, rot), and environmental problems (noise, pollution, inadequate space) (N. Smith & Middleton, 2007). Furthermore, household chaos and stress due to low-SES environments are associated with reduced parental attachment, harsh discipline, and poorer parental mental health (Bor et al., 1997; Costello et al., 2001; Sampson & Laub, 1994; J. R. Smith & Brooks-Gunn, 1997). While SES is often relatively narrowly defined within the literature (for example by only using parental income, education, or occupation as a proxy), it is becoming increasingly recognised that this is an oversimplification that does not account for the child’s individual experience and ignores variability in association with developmental measures depending on the precise SES measure (Lipina, 2017). An additional consideration is higher-SES sampling bias, which we attempted to avoid by testing all children within the target age range in participating schools. The downside of this approach is that it limited our SES measures to a child-friendly affluence index (based on available items and activities), and a postcode-based deprivation index (computed by the UK Government; it comprises income, employment, education, health, crime, barriers to housing and services, and living environment into a single rank score).

In addition to the many possible environmental factors that could impact educational attainment directly, there may be other developmental processes that influence it. One candidate is cognition. Relative to their peers, children from socio-economically disadvantaged backgrounds demonstrate lower performance on tests of attention (D’Angiulli et al., 2008; Kishiyama et al., 2009; Lipina et al., 2013; Mezzacappa, 2004; Neville et al., 2013; Stevens et al., 2009; Weatherholt et al., 2006), short-term memory (Farah et al., 2006; Kishiyama et al., 2009; Lipina et al., 2013; Noble et al., 2007; Sarsour et al., 2011), long-term memory (Farah et al., 2006; Noble et al., 2007), executive function (Farah et al., 2006; Kishiyama et al., 2009; Lipina et al., 2005, 2013; Noble et al., 2005, 2007), inhibition (Farah et al., 2006; Lipina et al., 2013; Noble et al., 2005; Sarsour et al., 2011; Sheridan et al., 2020), phonological awareness (Noble et al., 2005, 2006, 2007; Whitehurst, 1997), reading-related factors (Dolean et al., 2019; Noble et al., 2006; Sheridan et al., 2020), and general intelligence (Brito & Noble, 2014; Brooks-Gunn & Duncan, 1997; Gottfried et al., 2003; Hanscombe et al., 2012; J. R. Smith et al., 1997). Indeed, Sheridan and colleagues use a network approach to demonstrate that deprivation is tightly connected with cognitive performance, more so than with emotional reactivity measures (Sheridan et al., 2020). Importantly, although cognitive ability is partly hereditary, it is also impacted directly by environmental factors such as SES (Capron & Duyme, 1989; Hamadani et al., 2014; Hanscombe et al., 2012; Marcus Jenkins et al., 2013; Stumm et al., 2019; Turkheimer et al., 2003). It is thus a strong possibility that relative socio-economic disadvantage impacts educational attainment via a more direct influence on cognition.

A second possible mediator is mental health. Those who grow up in lower-income households are at greater risk of poorer mental health (HM Treasury, 2008; Lipman et al., 1994; Mayer, 2002; Meltzer et al., 2000) (see (Reiss, 2013) for a systematic review). This relationship is partly accounted for by various risk factors that are more likely to occur in low-income environments: family history of mental illness, reduced parental warmth, lack of supervision, and harsh punishment (Costello et al., 2001).

A further possible psychological mediator is attitude. With potential to exacerbate or mitigate the negative impact of lower SES on attainment, attitude is a plausible route through which heterogeneities in educational outcomes among children of similar SES could be shaped. Attitude measures previously demonstrated to mediate the effects of SES on educational outcomes include growth mindset (Claro et al., 2016), and big-five personality characteristics, including conscientiousness (Nieuwenhuis et al., 2015). Further attitude measures associated with academic outcomes, include grit (Duckworth et al., 2007) and school liking (Riglin et al., 2013).

### Previous Research

Taking action to mitigate the impact of relative socio-economic disadvantage on educational attainment requires that we understand the pathways of this influence. Progress has been slow because this requires a broad set of measures on the same individuals. For example, it is not uncommon for studies to use a simplified SES measure (income), one potential mediator (growth mindset), and a single outcome (averaged maths and reading score) (Claro et al., 2016).

Studies that go beyond using a single mediator tend to still be limited in the developmental categories they include because they are designed to address specific theoretical hypotheses. For example, measuring multiple attitude and school environment factors to explore the resilience-promoting features of schools (Borman & Overman, 2004), but to the exclusion of other factors outside this theoretical framework, such as cognition and mental health. This approach, while valuable for hypothesis testing, can overlook the potential complexity of inter-dependent mediating mechanisms between groups of variables, and ultimately reduces the possibility of identifying the best targets for intervention.

Another challenge for the field is methodological. Typical analysis methods do not account for the heterogeneity of outcomes among children from diverse socio-economic backgrounds. For example, many studies typically treat a particular SES bracket as a single group, or SES as a single continuous predictor. In reality, children respond to their environment in different ways. While children in longitudinal studies of the Kuaui sample who were in low-income environments at age 2 were more likely to develop problems with learning, behaviour, and mental health; one-third of the sample did not (Werner, 1989, 1993). This suggests that there may be relative vulnerability versus resilience to socio-economic adversity across children. Most studies do not employ analytic methods that would be able to capitalise on this heterogeneity.

### Current Study

As outlined above, the existing literature has explored direct relationships between a handful of variables at a time, often driven by specific theoretical frameworks. However, building a comprehensive understanding of the complex relationships between developmental factors requires a broad range of multiple different cognitive, environmental, and psychometric variables. The purpose of the current study was to collect and analyse such a broad dataset, by measuring educational progress, mental health, attitude characteristics, socio-economic variables, and multiple aspects of cognition, all within the same cohort of children. To do this, we developed a comprehensive assessment battery comprised of gamified tests and age-appropriate questionnaires for children aged 7–9 years (Bignardi et al., 2021). We used this to assess 519 children in their own classrooms in the East of England. We then employed data-driven analytical approaches that allowed us to incorporate this large and complex set of measures. This exploratory analytical approach is intentionally unconstrained by any single theoretical framework.

Many of studies cited above outline specific relationships, for example between SES and components of cognition (e.g. short-term memory or inhibition) or between “grit” and academic outcomes. Whether such relationships are indeed specific, depends greatly on whether the investigated variables systematically covary. For instance, if all cognitive components capture a common latent construct, then their individual relationships with SES are unlikely to be specific. The same holds true for grit, if this happens to be explained by a latent conscientious personality trait, then its association with SES is less likely to be specific. Our first goal was thus to identify the latent factors (“dimensions”) that underlie the wide range of variables that we collected.

Previous theoretical and empirical approaches have enforced a discrete subgrouping of children, characterised as “competence”, “resilience”, or “maladaptation” groups (Masten & Tellegen, 2012). Our second aim was to confirm whether such subgroups also emerge from the reverse (i.e. data-driven) approach. We thus employed cluster analysis to test whether discrete subgroups of children exist, on the basis of all their data.

The above approaches are excellent for identifying groups of latent factors or discretely different groups of participants. However, there is often more fine-grained detail in the relationships between variables, and reducing them to dimensions or groups can lose this fine-grained information. Our third aim was thus to complement the above approaches with a network analysis that maps at the intra-individual level the direct and indirect relations between socio-economic, educational, cognitive, attitude, and mental health variables.

With this study, we hope to acknowledge the complexity of the interrelated variables that characterise child development. Ultimately, mapping this constellation of variables should provide a comprehensive understanding of how these factors are associated, providing a firmer foundation for future mechanistic and intervention work, relative to designs in which just a few measures are included.

## Methods

### Sample and Recruitment

We aimed to recruit a sample that is representative of local socio-economic backgrounds. Schools were selected on the basis of having a high proportion of children with pupil premium status, which is a targeted UK policy to improve attainment for children from disadvantaged backgrounds. A total of 609 children from Years 3 and 4 (typically aged 7–9 years) were recruited from 22 classes from six different schools. At the time of testing, 88 children were not present due to e.g. illness, and 2 children were excluded for having over 20% missing data, resulting in a total of 519 usable datasets. Head teachers provided informed consent for their school’s participation, after which information letters were sent to parents’ home addresses. These included opt-out slips that could be returned to the school or the research team. At least four members of the research team visited schools after a minimum of two weeks from posting information letters, where they tested one classroom at a time. Children whose parents did object to testing were not tested. Our protocol was approved by the University of Cambridge Psychology Research Ethics Committee (PRE.2017.102).

### Power

The sample size was limited only by financial and practical feasibility. The usable datasets provide 80% power for traditional correlational analyses with effect sizes from R = 0.12 at α = 0.05. The sample size also affords enough power for cluster analyses, assuming a detectable distance between centroids (Δ = 4), and a minimum group size of 25 (Dalmaijer et al., 2020).

### Generalisability

The sample had an average age of 8.6 years (SD = 0.66, min = 7.3, max = 9.9), and an average z-transformed index of income deprivation affecting children index (IDACI; see under “*Deprivation index*” in the Supplementary Information) of −0.27 (SD = 0.94, min = −1.94, max = 2.29). While this index is described as a subset of the income deprivation score, it correlates very highly with the Index for Multiple Deprivation: R = 0.94, p = 3.80e-277. Thus, our sample was slightly less deprived than the general UK population, but the standard deviation and range indicate that it covered a representative variety of deprivation scores.

### Open Practices

The proposal for this study has been peer reviewed through the funder. The protocol and recruitment approach were reviewed by departmental committees and the local ethics committee. The analyses were not formally preregistered. Due to the sensitive nature of our participants and the inherent impossibility to anonymise deprivation data (reversing our transformations renders it a postcode-like localisation tool), we will release a synthetic dataset that has the same properties as the original. This supports reproducibility, but prevents potential de-anonymisation attempts. In addition, upon reasonable request and agreeing to refrain from attempting to identify any individual participants, fellow researchers will be allowed access to anonymised real data. All materials have been made available on https://github.com/esdalmaijer/2019_SES_child_development.

## Materials

We developed a custom tablet-based assessment that included both cognitive tests and age-appropriate questionnaires. Instructions in the assessment were both written and spoken, to avoid having to rely on children’s reading ability. Below is a general overview, and detailed information on each task can be found in the Supplementary Information, and in (Bignardi et al., 2021).

The application started with a general introduction that encouraged children to complete the assessment individually and explained volume adjustment. The running order of tasks and questionnaires was the same for all children with the most crucial measures completed earlier on, and engaging tasks interspersed among questionnaires with aim of maintaining attention and preventing boredom. The assessment, administered in the order reported here, consisted of two *educational performance* tasks which were a 3-min **reading fluency** task in which children had to read a sentence and indicate whether it was true or false, and a 3-min **sums** task to index maths fluency in which children had to perform age-appropriate additions, subtractions, and occasional multiplications and type their answer using an on-screen number pad (both similar to Woodcock-Johnson-III); a multi-target visual search task in which children had to find all 20 targets among 20 distractors within two minutes, from which **search organisation** (Benjamins et al., 2019; Dalmaijer et al., 2015) and **processing speed** (median inter-click times) were computed; a second visual search task, where discovered targets were not visually marked (and thus had to be remembered), was also used to compute processing speed (we did not compute search organisation indices for this task, because these are not validated for unmarked search tasks); questions on **household chaos** (Petrill et al., 2004), **family affluence** (Torsheim et al., 2016), **conscientious** (Barbaranelli et al., 2003), and **class distraction**; a digit span test to assess **verbal short-term memory** by asking children to briefly retain and reproduce increasingly long sequences of digits; followed by a rhyme judgement or a phonological awareness test (not incorporated into the current study due to it being changed midway through data collection); questions on **grit** (Furlong et al., 2013), general **anxiety** and **depression** symptoms (Muris et al., 2002), and **growth mindset**; a dot matrix test that assessed **spatial short-term memory** by asking children to briefly retain and reproduce increasingly long sequences of locations; a go / no go test of **inhibition** in which children had to quickly tap targets (80%) but avoid less common distractors (20%) that appeared at random locations on the screen; the series and classification sub-tasks of the Cattell Culture Fair test of **fluid intelligence** (Cattell, 1940); questions on **school liking** (Birch & Ladd, 1997; Ladd & Price, 1987), reading time, and technology usage, although we opted to exclude the latter two due to children’s assessment of time being highly inaccurate (e.g. frequent self-reports of over 24-h of activities per day); a general **number sense** assessment during each trial of which children had to assess which of two clouds consisted of more dots (Gebuis & Reynvoet, 2011; Odic & Starr, 2018); an evidence accumulation game (not reported on here due to being a non-standardised test included for experimental purposes); and a series of games that were intended as padding, to prevent children from finishing early and distracting others since completion time varied from approximately 40 min to an hour. Each of the tasks was preceded by a child-friendly and spoken instruction, and most tasks had interactive practice trials. For further details, see “*Tasks*” and *“Questionnaire”* in the Supplementary Information.

Questionnaire items were either from full questionnaires, or from subcomponents of wider questionnaires. Their origins are cited above, and a full list of items is included in Supplementary Table S1. We did not test whether our recombination of existing questionnaire items impacted the reliability of validity of these items.

Children were tested in their own classroom. Each child received a tablet computer that was preloaded with the assessment as described above, and connected to a headset so that they could listen to sounds and the spoken instructions. At least four members of the research team invigilated, and answered questions where necessary. (This occurred infrequently, and was mostly in response to questions about the meaning of words from questionnaire items, like “appetite”.) Privacy screen-covers and headphones ensured children could not see or hear each other’s tablet computers.

### Reliability and Validity

The assessments used here were based on well-established versions, although their self-paced delivery via tablet computer was more novel. In separate work (Bignardi et al., 2021) we demonstrated that our tasks are of moderate (inhibition) to good (reading fluency, sums/maths fluency, visual search, processing speed) reliability in the same sample described here, and an additional sample of individually tested children. Internal consistency could not be computed for the verbal short-term memory and spatial short-term memory tasks, due to their termination rules. Our tests were also of good predictive validity. Individual tests showed moderate to high correlations (*r* = 0.38 to 0.63) with teacher ratings of academic ability (with the exception of inhibition, *r* = 0.18). In addition, multivariable regression revealed that proxy measures of educational performance (reading and sums/maths fluency) explained 44.9% of variance in teacher ratings of academic ability (Bignardi et al., 2021).

In addition to the tablet assessment, we obtained children’s postcodes from their schools. We cross-referenced these with data from the UK government to obtain an index of deprivation for each child’s neighbourhood (see “*Deprivation index*” in the Supplementary Information).

### Analysis

We pre-processed and analysed our data in a custom Python pipeline that standardised data within each measure, imputed missing values using a 9-nearest neighbour algorithm trained on the 397 full datasets, and then executed the analysis described in brief below. For more detailed descriptions, please refer to the Supplementary Information.

### Dimensions and Clusters

To uncover the dimensions along which children vary, we employed principal component analysis with orthogonal (varimax) rotation on 19 measured variables. Parallel analysis was conducted to determine the significance of resulting components (see “*Principal component and parallel analyses*” in Supplementary Information).

Another approach to the concept of vulnerability versus resilience is to focus on the potential existence of discrete subgroups who respond differently to socio-economic disadvantage (Masten & Tellegen, 2012). We employed k-means clustering (Lloyd, 1982) after projecting our high-dimensional data into two dimensions using multi-dimensional scaling (Kruskal, 1964) to avoid the “curse of dimensionality” (Bellman, 1957). The k-means algorithm randomly generates k centroids, and iteratively updates these as the average of observations nearest each centroid, until a stable solution is reached. Silhouette coefficients were computed from the ratio between the distances between a sample, its assigned centroid, and the centroid of the closest cluster that it was not assigned to (Rousseeuw, 1987). Silhouette coefficients of all samples were averaged to compute a marker of overall quality of each solution, with values over 0.5 being considered evidence for the presence of clusters within a dataset (Kaufman & Rousseeuw, 1990).

### Network Analysis

Network analysis provides a way of unpicking complex relationships between many collinear variables (Epskamp et al., 2018). While factor analysis focusses on general underlying dimensions and clustering approaches on characterising sub-groups of individuals, here we investigated the relationship between measured variables (“deprivation”, “depression”, “inhibition”, etc.) by casting them as nodes that are connected via edges. The advantage of this *psychological network analysis* is that it can clarify *how* factors relate to each other (Borsboom & Cramer, 2013; Fritz et al., 2019; Schmittmann et al., 2013), without assuming directionality.

We employed multi-dimensional scaling (Kruskal, 1964) on the transposed dataset (casting 519 observations into two dimensions) to determine the positions of nodes. This resulted in a feature space in which nodes that appear more closely together were responded to more similarly by participants. As this was for illustrative purposes, no goodness-of-fit measures were computed.

We then computed edge weights through partial correlation, which quantifies the uniquely explained variance of one factor on another. To distinguish between meaningful and spurious partial correlations, we employed LASSO regularisation (Tibshirani, 1996), which sets spurious correlations to 0. What is considered a spurious correlation depends on a tuning parameter that we set through 5-fold cross validation. A more conservative approach was also implemented through 3000 iterations of bootstrapping (Efron, 1979), to estimate the probability of each edge occurring, and the variability of each edge’s strength. We calculated in how many iterations each possible edge appeared, and compared this against the expected number of edges to occur by chance, defined as the 95th percentile of the number of edges observed in all iterations, or the Bonferroni-corrected 99.97th percentile. We considered edges that appeared in more iterations than chance to be statistically significant. We also computed 95% confidence intervals of edge weights, defined as the 2.5th and 97.5th percentile of estimated partial correlations (Epskamp et al., 2018) in all bootstrapping iterations.

## Results

### Descriptives

Means, standard deviations, and ranges (minimum and maximum) for variables used in subsequent analyses are provided in Table 1. These reflect the (sub-)scores for variables before standardisation.

**Table 1:** Means (M), standard deviations (SD), and minimum-maximum ranges (min, max) for scores associated with each variable used in later analyses. Abbreviations: “AU” arbitrary units, “STM” short-term memory, “Sums” maths fluency

	M	SD	min	max
**Affluence** (AU)	4.95	3.11	−5.00	12.00
**Anxiety** (RCADS, AU)	2.15	1.32	0.00	5.00
**Calm home** (AU)	0.74	1.09	−3.00	3.29
**Class distraction** (AU)	0.51	0.32	0.00	1.00
**Conscientiousness** (AU)	1.58	0.77	0.00	3.00
**Depression** (RCADS, AU)	1.79	0.90	0.00	5.00
**Deprivation** (IDACI, z-scored rank)	−0.27	0.94	−1.94	2.29
**Fluid reasoning** (Cattell series, number correct)	5.80	2.79	0	12
**Fluid reasoning** (Cattell classification, number correct)	5.91	1.95	1	11
**Grit** (AU)	2.87	0.80	0.36	4.00
**Growth mindset** (AU)	0.80	0.28	0.00	1.00
**Inhibition** (Go/NoGo, d’)	3.13	0.93	−3.26	4.95
**Number sense** (proportion correct)	0.63	0.09	0.32	0.86
**Reading** (number correct)	45.24	13.52	4	84
**School liking** (AU)	0.04	0.65	−1.00	1.00
**Search** (marked search task, best R)	0.61	0.20	0.02	0.99
**Search** (marked search task, intersect rate)	0.18	0.13	0.00	1.17
**Search** (marked search task, pixel distance between identified targets)	326.24	46.03	240.76	770.39
**Spatial STM** (number correct)	5.94	4.11	0	16
**Speed** (marked search task, seconds between identified targets)	0.63	0.28	0.30	4.28
**Speed** (unmarked search task, seconds between identified targets)	0.61	0.35	0.23	6.93
**Sums** (number correct)	34.47	14.36	0	85
**Verbal STM** (number correct)	9.48	4.12	0	20

### Principal Component Analysis Reveals 3–5 Dimensions

Five principal components accounted for 52.41% of the total variance, and the 5-factor solution (after varimax rotation) accounted for 38.19% of the factor’s common variance. Table 2 outlines the explained variances per factor (hereafter named “rotated components”).

**Table 2:** Factor loadings after orthogonal (varimax) rotation. All components had eigenvalues over 1, but only those indicated in bold were identified in a parallel analysis. Bold values indicate the highest factor loading for each variable. Factor names were generated by the authors on the basis of their interpretation of factor loadings. Abbreviations: “RC” rotated component, “SES” socio-economic status, “STM” short-term memory, “Sums” maths fluency

	RC 1	RC 2	RC 3	RC 4	RC 5	
	*Cognition*	*Attitude*	*Mental health*	*Speed*	*SES*	*Uniqueness*
Affluence	0.071	0.017	0.029	0.050	**0.491**	0.751
Anxiety	−0.075	−0.125	**0.563**	0.010	−0.016	0.661
Calm home	0.017	**0.438**	−0.173	0.003	0.085	0.770
Class distraction	−0.213	**−0.340**	0.335	0.064	−0.021	0.722
Conscientiousness	−0.038	**0.655**	0.045	0.030	−0.066	0.562
Depression	−0.206	−0.222	**0.776**	−0.007	−0.077	0.300
Deprivation	−0.217	−0.059	0.120	0.035	**−0.375**	0.793
Fluid reasoning	**0.566**	0.103	−0.179	0.000	0.221	0.588
Grit	0.154	**0.604**	−0.016	0.060	0.198	0.568
Growth mindset	0.132	**0.445**	−0.157	0.040	0.047	0.756
Inhibition	**0.452**	0.106	−0.062	−0.007	−0.134	0.762
Number sense	**0.569**	0.051	−0.059	0.059	0.099	0.656
Reading	**0.506**	0.062	−0.047	0.212	0.145	0.671
School liking	0.131	**0.524**	−0.195	−0.030	−0.109	0.658
Search	**0.269**	0.055	−0.075	0.206	−0.010	0.877
Spatial STM	**0.666**	0.095	−0.034	0.020	0.057	0.542
Speed	0.211	0.030	0.089	**0.960**	0.044	0.023
Sums	**0.727**	0.067	−0.094	0.149	0.171	0.406
Verbal STM	**0.483**	0.073	−0.153	−0.017	0.247	0.677
**Eigenvalue** (before rotation)	4.22	2.12	1.38	1.20	1.06	
**Explained variance** (%)	22.17	11.11	7.24	6.32	5.56	
**Sum of squared loadings**	2.63	1.69	1.23	1.05	0.65	
**Explained common variance (%)**	13.86	8.92	6.48	5.53	3.40	

Three components were identified by parallel analysis (Horn, 1965). The first component was labelled “cognition” due to its loadings on sums/maths fluency, spatial short-term memory, approximate number sense, fluid reasoning, reading fluency, verbal short-term memory, inhibition, and search organisation. The second component was labelled “attitude” due to its loadings on conscientiousness, grit, school liking, and growth mindset, and class distraction; with an additional loading on home calmness (admittedly not directly an “attitude” variable). The third component was labelled “mental health” due to its loadings on depression and anxiety.

A further two components were not apparent in the parallel analysis, but had eigenvalues over 1 (and thus abide by Kaiser’s criterion). These were labelled “speed” due to a loading on processing speed; and “socio-economic status” due to loadings on affluence and deprivation.

Note that all task measures and no questionnaire measures load onto the first component, with the exception of speed and search organisation which are both derived from the visual search task. While the sub-grouping of questionnaire measures into seemingly meaningful components suggests this analysis is not solely driven by measurement type, we make two suggestions: (1) we emphasise the need for network analyses which go beyond the relatively coarse clumping of variance; (2) we advise caution in the interpretation of our derived components, as one cannot distinguish between the presence of broad underlying statistical entities, or a series of more nuanced relationships between individual measures.

### Cluster Analysis Reveals no Discrete Sub-Groups

We found no evidence of discrete clusters: average silhouette coefficients were just under 0.4 for all solutions from k = 2 to k = 10 (Supplementary Fig. S1), whereas the accepted threshold for a robust cluster structure is at 0.5. These results suggest it is unlikely that discrete subgroups with specific vulnerability or resilience properties exist in the sample.

### Network Analysis of the Relationships between all Variables

**Fig. 1.**
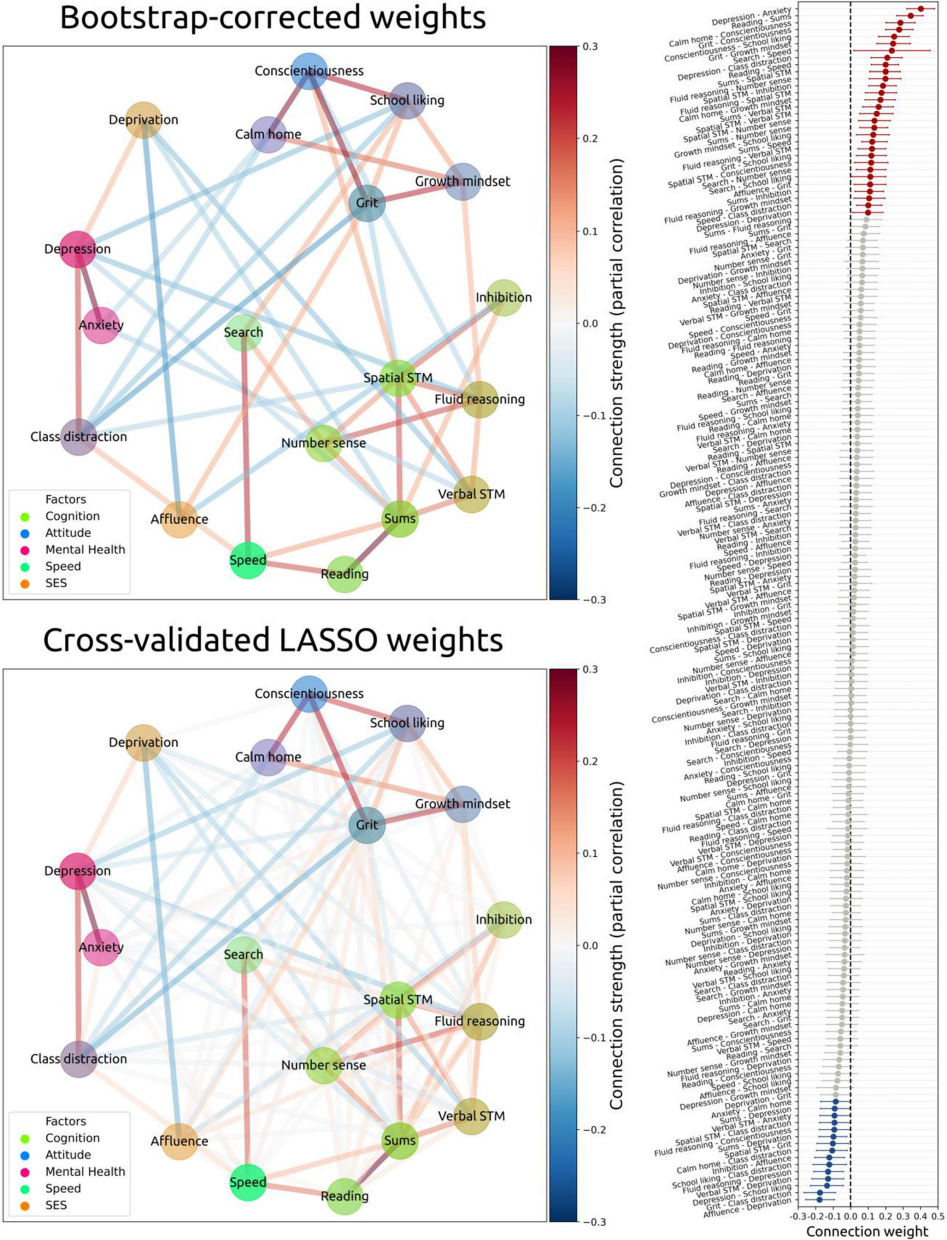
(next page) – Network analyses that describe the relationships between variables measured in 519 children aged 7–9 years. Nodes are coloured according to principal components: **cognition** (chartreuse), **attitude** (azure), **mental health** (rose), **speed** (spring green), **socio-economic status** (orange). Node positions are determined through multi-dimensional scaling, and edge weights through partial correlation. Spurious partial correlations are filtered through bootstrapping (top-left panel) or LASSO regularisation with parameter selection through 5-fold cross-validation (bottom-left panel). All possible connections and their bootstrapped 95% confidence interval are listed in the right panel. Confidence intervals that include 0 (grey) were considered not statistically significant.

**Fig. 2:**
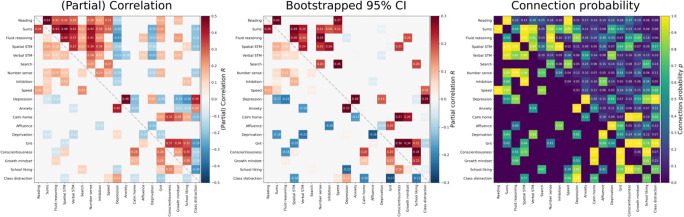
The left panel shows correlations that are statistically significant after Holm-Bonferroni correction (top-right triangle), and partial correlations (bottom-left triangle) between all measured variables. The middle panel shows high (top-right triangle) and low (bottom-left triangle) bootstrapped 95% confidence intervals for the partial correlations, but only those that do not include 0. The right panel shows connection probabilities estimated through bootstrapping (top-right triangle), and only those that are higher than chance (bottom-left triangle)

Node positions and connection strengths were determined through bootstrapping (Fig. 1, top-left panel) or through LASSO regularisation with cross-validated tuning parameter (Fig. 1, bottom-left panel). We considered two forms of statistical significance. The first is when a connection’s 95% confidence interval did not include 0 (Fig. 1, right panel; Fig. 2, middle panel). The second was a binarised version, in which a connection was considered statistically significant if it occurred in more iterations than chance. Uncorrected chance was set as the 95th percentile (α = 0.05) of the number of connections found in all iterations (Fig. 2, right panel), i.e. 65 connections (38% of iterations). This was Bonferroni-corrected for multiple comparisons by setting chance at the 99.97th percentile (α = 0.0003), which translated to 73 connections (42.7% of iterations). The bootstrap-corrected network (Fig. 1, top-left panel) connection probabilities ranged from 0.54 to 1, meaning they survived binarised Bonferroni-correction.

Variables are colour-coded (Fig. 1) according to their loadings onto each of the 5 components derived from the principal components analysis: **cognition** (chartreuse), **attitude** (azure), **mental health** (rose), **speed** (spring green), **socio-economic status** (orange). The positioning of the nodes resembles the results of the principal components analysis, corroborating that the components capture similarity in construct and not just metric type since commonality among many variables due to shared measure type is partialled out of the network results.

Direct connections existed between socio-economic variables and all other thematic categories: deprivation related to verbal short-term memory, sums/maths fluency, and depression symptoms; and affluence related to inhibition and grit. Another environmental variable was home calmness (reversed household chaos), despite it loading primarily on the “attitude” component. This related to conscientiousness, growth mindset, class distraction, and anxiety symptoms. With two exceptions among sums/maths fluency’s connections (with depression and deprivation), educational outcomes were exclusively directly related to cognitive factors: sums/maths fluency related to spatial and verbal short-term memory, number sense, processing speed, inhibition, and fluid reasoning; and reading fluency related to processing speed. Mental health factors, while correlated with educational outcomes, only showed a single direct relationship: between sums/maths fluency and depression. Depression symptoms were directly related to class distraction, school liking, and fluid reasoning; and anxiety symptoms were directly related to verbal short-term memory. A similar pattern was observed for attitude-related factors, which were correlated with educational outcomes, but did not show any direct relationships in the network. Instead, conscientiousness and grit related to spatial short-term memory, and both conscientiousness and growth mindset related to fluid reasoning. Grit also related to class distraction, and conscientiousness and growth mindset showed relations to school liking, as well as to having a calm home. Class distraction and school liking related to each other, and to cognitive factors: class distraction to spatial short-term memory and processing speed; and school liking to search organisation.

In sum, cognition played a central role as the pivot point between educational outcomes, mental health, and attitude. Crucially, socio-economic status (affluence and deprivation) directly related to all thematic groups of variables (cognition, attitude, mental health, and educational outcomes).

## Discussion

We developed a tablet-based assessment (Bignardi et al., 2021), and used this to collect data from 519 children aged 7–9 years in the East of England. Using exploratory factor analysis, we found three latent factors underlying our measures: cognition, attitude, and mental health (parallel analysis); and a further two that only met Kaiser’s criterion: speed and socio-economic status. Cluster analysis provided no evidence for discrete sub-groups of “resilient children” among our sample. By measuring a wide array of variables, we could map their interrelatedness in a network analysis: socio-economic status had direct associations with all categories of developmental variables; and cognitive development was closely related to educational progress (sums/maths and reading fluency), and the hub through which variables associated with children’s mental health and attitudes indirectly related to educational progress.

Discrete subgroups, by definition, are groups of individual observations that do not show any overlap. Non-overlap is an assumption in many clustering methods, and is sometimes made very explicit (e.g. in Tibshirani et al., 2001). This does not mean that partially overlapping groups are not different: the traditional Cohen’s d effect sizes in Psychology of group differences that are small (0.3), medium (0.5), or large (0.8) correspond to an overlap between the groups of 88, 80, and 69%, respectively. What constitutes a “cluster” is a matter of philosophical (Hennig, 2015) and methodological (Dalmaijer et al., 2020) debate, and one could argue that partially overlapping distributions could also reflect distinct subgroups. However, in the context of resilience to socio-economic disadvantage, it is important to note that the identification of “subgroups” was on the basis of cutoff scores (Masten & Tellegen, 2012). Here, when searching for subgroups with the appropriate data-driven methodology, no distinct groups emerge. This null finding does not evidence the absence of heterogeneity in response to socio-economic disadvantage along continuous dimensions, but rather the absence of discrete (or sufficiently distanced but partially overlapping) subgroups.

The literature to date primarily comprises studies of relationships between one or a few measures and children’s environment. For example, showing that socio-economic status is linked to mental health (Reiss, 2013), attitude (Chapman et al., 2010), or educational outcomes (Andrews et al., 2017). Few studies have attempted to explore how different factors might interact, for instance highlighting the buffering effect of personality characteristics on the relationship between socio-economic status and educational attainment (Nieuwenhuis et al., 2015), and the interactions between socio-economic status, phonological awareness, short-term memory, and reading (Dolean et al., 2019).

Crucially, there was little evidence for a direct relationship between mental health or attitude and educational outcomes. Instead, their impact is primarily indirect, through cognitive development. In short, while poor mental health and attitudes are predictive of children’s educational attainment (Duckworth et al., 2007; Johnston et al., 2014; McLeod & Kaiser, 2004; Veldman et al., 2014), our results suggest these pathways are largely through cognition. For example, Claro et al. (2016) conclude that growth mindset tempers the negative effect of low SES on academic achievement; our network corroborates this finding but indicates the relationship is more complex when accounting for a broader range of variables, with affluence being related to growth mindset indirectly through grit, and in turn, growth mindset influencing sums/maths fluency via direct and indirect associations with various cognitive measures.

In adults, poor mental health has a negative impact on cognitive functioning in those with anxiety (Eysenck et al., 2007) or depression (Thomas & O’Brien, 2008). Indeed, “diminished ability to think or concentrate” is a diagnostic criterion for depression (American Psychiatric Association, 2013) that occurs in three quarters of moderately depressed adults (Tolentino & Schmidt, 2018). Similar negative effects of anxiety and depression on cognition have been reported in children (Günther et al., 2004), but not consistently (Mayes & Calhoun, 2007). Our results confirm that sub-clinical anxiety and depression symptoms directly relate to cognitive variables (verbal short-term memory and fluid reasoning), which in turn relate to sums/maths fluency; and even one direct link between depression and sums/maths fluency. These findings bolster confidence in mental health support as a potential intervention for struggling learners.

Being of a higher socio-economic status increases the likelihood of being more conscientious in adulthood (Chapman et al., 2010), and to display a growth mindset in high-school students (Claro et al., 2016). Additionally, big-five personality characteristics buffer negative effects of neighbourhood disadvantage in adolescents (Nieuwenhuis et al., 2015). Our results confirm affluence relates to attitude, specifically grit in our analysis, which in turn is strongly directly related to conscientiousness and growth mindset. It is important to note that measures of grit and growth mindset are not unbiased with regard to SES, and that growth mindset’s supposed relationship to educational achievement only holds in students (here about 15–16 years) from higher-SES families (King & Trinidad, 2021).

These attitude variables relate to spatial short-term memory and fluid reasoning, and it is only via these mediating relationships that attitude relates to educational outcomes. Specifically, conscientiousness positively relates to spatial short-term memory, and negatively to fluid reasoning; grit relates negatively to spatial short-term memory; and growth mindset positively relates to fluid reasoning. We recommend not over-interpreting this specificity (see next paragraph), and can only offer speculative post-hoc explanations of why direct connections exist between attitude and cognitive variables. Positive relationships could be due to the fact that it might be easier to develop a positive attitude towards homework (a central topic in questions on grit and conscientiousness) for children for whom homework is easier due to better cognitive abilities. In reverse, improving attitude could be a compensatory mechanism for those with lower cognitive abilities. In our cross-sectional data, it is impossible to confirm either of these speculations.

Importantly, grit and conscientiousness questionnaires contain similar items, and potentially measure the same construct. This is supported in our results by conscientiousness, grit, and growth mindset all loading on one latent factor (Table 2), and their proximity and strong direct relations in our network analysis (Fig. 1). These findings align with those reported in high school pupils, for whom grit is not predictive of educational outcomes after accounting for conscientiousness (Ivcevic & Brackett, 2014). In university students, grit overlaps with conscientiousness, self-control, and growth mindset (Hwang et al., 2018; Kannangara et al., 2018). While the concept of “grit” is considered to be different from conscientiousness due to the inclusion of long-term goals (Duckworth et al., 2007), there is considerable conceptual and quantifiable overlap, and our results indicate attitude does not operate independently from socio-economic and cognitive variables.

While the existing literature emphasises the unique contributions of grit (Eskreis-Winkler et al., 2014) or growth mindset (Claro et al., 2016), our findings advocate a more holistic and broader consideration and measurement of children’s attitudes and cognitive development. Of potential practical value for teachers is that both class distraction and school liking relate directly to mental health, attitude, and cognitive factors. Thus, easily observed changes in school liking and class distraction are promising markers of children who are more likely to struggle. Ultimately, it is only through understanding the constellation of developmental factors that we can derive targets for meaningful educational policies and interventions.

### Limitations

One limitation of this study is its cross-sectional nature, impeding causal conclusions. While it seems likely that deprivation causes poorer cognition and educational outcomes (rather than vice versa), other pathways are harder to disentangle. For example, it could be that poor mental health leads to poorer cognition and in turn to poorer educational outcomes; but alternatively poorer educational progress could impact upon cognition, which in turn could be a risk factor for poorer mental health. Longitudinal data from this and other cohorts is necessary to disentangle these relationships.

Another potential limitation is our reliance on tablet-based measures to infer educational progress. Real educational data is very coarse at this stage of primary school, with children being categorised as either below, at, or above age-expected levels. However, teacher ratings of children’s ability could provide higher-resolution insight. While we opted not to rely on this measure due to concerns regarding potential biases in teacher ratings, we found strong correlations (0.55 and 0.63) between teacher ratings and our sums/maths and reading fluency measures (Bignardi et al., 2021). We are thus confident our assessment taps into real educational progress.

A further limitation is that our developmental measures are inherently a combination of both performance and attention. Factors including interest, fatigue, frustration, and momentary motivation will all contribute to the attention a child devotes to a task or questionnaire. While we attempted to mitigate the effects of boredom by distributing more engaging tasks among the less interesting questionnaires, individual children will have individual preferences as to what tasks they consider engaging.

While we stressed the importance of broadening the definition of SES in the Introduction, we relied on two measures that relate heavily to income. Specifically, we used an affluence questionnaire that asked about possessions and activities that require sufficient income, and we used a neighbourhood income deprivation index (although it correlates R = 0.94 with the supposedly wider Index of Multiple Deprivation). This is a limited index of SES, and our results further illustrate this: SES variables were related to variables in the cognition, attitude, and mental health domains (Fig. 1), and particularly deprivation had a broad pattern of correlation (Fig. 2, left panel). In other words, measures of cognition or depression can also be indirect measures of SES. This is a methodological problem, as it complicates making a practical distinction between SES and non-SES variables; but it could also reflect on theory: should some of the variance in e.g. mental health be considered a core socio-economic issue?

## Conclusion

In a large sample of primary school children aged 7–9 years in the East of England, variability in a broad assessment of educational outcomes, cognition, mental health, attitude, and socio-economic status is captured by cognitive ability, attitude, and mental health components. While there is variability in outcomes, we did not find this to be reflected in discrete sub-groups of children characterised as being “vulnerable” or “resilient” to the effects of socio-economic status. The network structure of developmental measures reveals educational progress is directly associated with mental health, socio-economic status, and cognitive development, but not attitude factors. Cognition also broadly affords further indirect relations between educational progress and all other factors. Furthermore, we demonstrate that socio-economic variables have widespread direct detrimental relations to cognition, mental health, educational progress, and attitudes; and further indirect consequences due to the entanglement of these variables. Finally, we highlight findings which, with sufficient confirmation from other studies, could prove plausible the identification and support of children who are struggling: classroom distraction and school liking are potential markers for teachers to identify those children experiencing poorer educational progress, and mental health support could be an effective intervention to support children who are falling behind.

## Supplementary Information

ESM 1 (PDF 4.01 MB)

## Data Availability

Due to the sensitive nature of our participants and the inherent impossibility to anonymise deprivation data (reversing our transformations renders it a postcode-like localisation tool), we will release a synthetic dataset that has the same properties as the original. This supports reproducibility, but prevents potential de-anonymisation attempts. In addition, upon reasonable request and agreeing to refrain from attempting to identify any individual participants, fellow researchers will be allowed access to anonymised real data. All materials have been made available on github.com/esdalmaijer/2019_SES_child_development.
