# Supplementary material for: Direct and indirect links between children’s socio-economic status and education: pathways via mental health, attitude, and cognition
Source: Curr Psychol. 2021 Sep 4;42(12):9637–51. doi: 10.1007/s12144-021-02232-2 (PMC7614555; doi:10.1007/s12144-021-02232-2)
Supplement: Supplementary file 1 — (PDF 4.01 MB) [file 12144_2021_2232_MOESM1_ESM.doc]

Supplementary Information

Direct and indirect links between children’s socio-economic status and education: Pathways via mental health, attitude, and cognition

[BLINDED]

*[BLINDED]*

**Corresponding author**

[[](mailto:edwin.dalmaijer@mrc-cbu.cam.ac.uk)BLINDED]

# Supplementary Methods

## Tasks

### Reading fluency

For a duration of three minutes, children were presented with sentences, and for each they indicated whether it was true by clicking on one of two buttons (“yes” or “no”). The sentences were simple, unambiguous, and had a clear correct answer. Examples include: “A dog can fly”, “The number ‘5’ is a letter”, “A fork can be used for eating”, and “Birds can lay eggs”. The outcome measure of interest for this task was the number of correct answers within 3 minutes. Intending to prevent ceiling effects, we used a total of 101 sentences, so that only those able to respond at a pace of 1 sentence per 1.78 seconds would be able to complete the entire set.

It should be noted that this task can be “gamed” by rapidly clicking on a random button, because this also increases the number of correct answers. However, this type of behaviour would also reduce the proportion of correct items. In our sample, under 6% of children answered less than 70% of sentences correctly. This indicates that the vast majority of children performed the task as intended.

### Maths fluency

For a duration of three minutes, children were presented with basic sums that entailed addition, subtraction, and a few multiplications. All sums consisted of two term, each in the range 0-10. The correct outcomes were always positive, and generally 10 or lower (out of 159 sums, 53 had a result over 10). The task became generally harder, with multiplications and higher outcomes being more likely towards the end of the task. Responses were given via an on-screen number pad for the numbers 0-9, a backspace button, and a confirm button. The outcome variable of interest was the number of correctly answered sums within 3 minutes.

### Verbal short-term memory

We employed a forward digit span task, in which children hear and see three or more digits. They were asked to remember these digits in order. After seeing and hearing all digits, and a 1 second delay, children were asked to reproduce the digits using a keypad with the numbers 0-9 and a backspace button. Once the current number of digits was typed in, the next trial started. The task started at three digits per trial, and progressed to a maximum of nine. For each possible span, six trials were presented, and the task stopped progressing when three or more trials within a span were answered incorrectly. The outcome measure of interest was the number of correct trials.

### Spatial short-term memory

The spatial short-term memory task was very similar to its verbal counterpart, and was modelled after the well-known dot matrix task. In this task, children see a 4x4 grid in which dots appear sequentially. After 1 second, children could reproduce the sequence by tapping the cells in which dots appeared, in the same order as they were presented. Before starting the task, children watched a video of an example trial and correct response. The task started at three dots per trial, and progressed to a maximum of nine. For each possible span, six trials were presented, and the task stopped progressing when three or more trials within a span were answered incorrectly. The outcome measure of interest was the number of correct trials.

### Fluid reasoning

To assess children’s fluid reasoning skills, we employed the Series and Classification sub-tests from Cattell’s Culture Fair test (Cattell, 1940). The Series task presents children with three abstract figures and an empty fourth box. Children are asked which from a selection of five further drawings would fit the empty box best, with the aim to further the series in a logical way. The task was preceded with three examples, and ran for 12 progressively more difficult trials or until a time limit of 3 minutes was passed. The Classification task presents children with five abstract figures that share features, and asks children to find the figure that is different in some way. The task was preceded with two examples, and ran for 14 progressively more difficult trials or until a time limit of 4 minutes was passed. Children selected options by tapping them, and could change their mind after initial selection by clicking on a different option. They could then click a “confirm” button to continue to the next trial. The outcome measure of interest was the number of correct trials in each task.

### Number sense

The number sense task was designed to test the “approximate number system”: the general ability to estimate quantities that some argue is paramount for mathematical cognition (for a more thorough introduction, see (Odic & Starr, 2018)). Number sense was assessed using non-symbolic numerical representations, specifically clouds of dots. To decouple numerosity and surface area, we generated stimuli that were composed of dots of different sizes and that were more or less dense, using a toolbox developed specifically for this purpose by (Gebuis & Reynvoet, 2011). In each of 116 trials, we presented one dot cloud on the left and one on the right. Within 3 seconds, children had to indicate which cloud contained more dots. The trials had dot number ratios 1.1, 1.2, 1.3, 1.5, and 2. The inter-trial interval was 0.5 seconds. The task started with three example trials that had no time limit, and that were always followed by an explanation of what the correct answer was. The outcome measure of interest was the proportion of correct trials.

### Multi-target visual search

We employed a cancellation task, in which children were asked to find and tap 40 targets among 40 distractors. Targets were smiling cat emoticons, and distractors were visually similar neutral cat emoticons. Stimuli were presented on a jittered grid in which targets were uniformly distributed across columns. When tapped, targets produced a “meow” sound, whereas distractors did not. This task is a child-friendly adaptation of a cancellation test used in neurological assessment (Dalmaijer et al., 2018; Malhotra et al., 2006; Parton et al., 2006). In the first iteration of the task, targets were marked with a red cross after being pressed. In the second, a different stimulus array was used, and this time targets were not visually marked. Children thus had to memorise the locations they had tapped to successfully complete the task. Each of the tasks started with a single practice cancellation in which children could tap a target and a distractor, and received spoken feedback when tapping either. The button to start the task did not appear until the practice target was tapped.

One outcome measures of interest related to **search organisation**, which was quantified as the composite score of the standardised outcomes of the “best R” (the correlation between click rank order and horizontal or vertical position), intersect rate (number of times a search path crosses itself divided by the number of target clicks in that path), and the average distance between consecutive target clicks from the task with visible markings. The second outcome measure of interest related to **processing speed**, and was computed as the median time between two consecutive clicks in both iterations of the task (standardised within each task, and then averaged). Further details on the rationale and computation of these metrics can be found in (Dalmaijer et al., 2015).

### Inhibition

We employed a Go / No Go task to test children’s inhibition. In our version of this task, children were presented with one stimulus at a time, 80% of which were targets (dog emoticons) that had to be tapped, and 20% were distractors (smiling turd emoticons) that children had to avoid tapping. Upon tapping a target, children heard a barking sound and were awarded 50 points. Upon clicking a distractor, children heard a fart noise, and 150 points were subtracted from their total. Only one stimulus was visible at a time, for 1.5 seconds or until it was tapped, after which a delay of 0.3-0.8 seconds occurred. The task lasted 4 minutes.

The outcome measure of interest was the sensitivity index (d’), a signal-detection metric that quantifies how well children could separate targets from distractors. High values of d’ were assigned to children who were high on hits (clicked targets) and low on false alarms (clicked distractors). This measured how inclined children were to click on the distractor while taking into account target-clicking behaviour, thereby preventing children who adopted a strategy of not clicking on many stimuli (target or distractor) to be marked as excellent inhibitors.

## Other data

### Deprivation index

The United Kingdom’s government computes and publishes an index of multiple deprivation for every lower-layer super output area in England (Department for Communities and Local Government, 2015). There are 32844 of these areas in England, each with an average of 1500 residents. The index of multiple deprivation combines income, employment, level of education, health, crime, barriers to housing and services, and living environment within each area to produce a rank order. In addition to this general deprivation index, the UK government computes an index of income deprivation affecting children (IDACI), which we used in the current study.

Schools provided postcodes for each included child, on the basis of which we determined the IDACI score of the associated area. We transformed the IDACI rank score to a z-score by subtracting the rank from the maximum rank value (32884), then dividing it by the maximum, and submitting that to an inverse normal (μ=0, σ=1) distribution (percent point) function (Bishop, 2018).

### Questionnaire

We included child-friendly questionnaires in our application that were presented as “quizzes” (Supplementary Table S1). We included questions on **depression and anxiety symptoms** from RCADS (Muris et al., 2002). We also included items from the household chaos questionnaire (Petrill et al., 2004) to measure **home calmness**, and from the revised family **affluence** questionnaire (Torsheim et al., 2016). Furthermore, we included questions from a series of attitude-related questionnaires, including on **conscientiousness** (Barbaranelli et al., 2003), **grit** (Furlong et al., 2013), and **growth mindset**. Despite the questions being highly similar in topic, here we present them as different constructs, in line with current developmental psychology literature. We highlight the questions on **school liking** and **class distraction**, because we consider these consequences of mental health and attitude rather than direct reflections of attitudes. The growth mindset question “You have a certain amount of talent, and you can't do much to change it” was eliminated from further analysis, as it was barely correlated with the core growth mindset question “You can always become more clever”, likely due to it being phrased in a way that is likely to confuse 7-9 year old children.

**Supplementary Table S1** – Questions from the quizzes in our tablet-based assessment. A ‘+’ in the weight column indicates that a question contributed positively to the construct, and a ‘-’ that it contributed negatively. Construct scores were computed as weighted sums.

| **Construct** | **Weight** | **Question** |
| --- | --- | --- |
| **Depression** | + | I feel that nothing is much fun anymore. |
|  | + | I feel sad or empty. |
|  | + | I feel very tired. |
|  | + | I feel like I don’t want to move. |
|  | + | I have problems with my appetite. |
| **Anxiety** | + | I worry about bad things happening to me. |
|  | + | I am worried that something bad will happen to myself. |
|  | + | I worry about what will happen. |
|  | + | I am worried that something awful will happen to my family. |
|  | + | I think about death. |
| **Calm home** | + | I have a regular bedtime routine. |
|  | + | The atmosphere in our house is calm. |
|  | + | Our home is a good place to relax. |
|  | + | Our home is quiet. |
|  | - | You can’t hear yourself think in our home. |
|  | - | There is usually a television turned on somewhere in our home. |
|  | - | Our home is messy. |
| **Affluence** | + | How many computers (PCs, Macs or laptops) does your family own? |
|  | + | How many times did you travel abroad for holiday last year? |
|  | + | How many bathrooms (room with a bath) are in your home? |
|  | + | Does your family own a car, van or truck? |
|  | + | Do you have your own bedroom for yourself? |
|  | + | Does your home have a dishwasher? |
| **Grit** | + | I finish all my class work. |
|  | + | When I get a bad grade, I try even harder the next time. |
|  | + | I keep working and working until I get my schoolwork right. |
|  | + | I do my class assignments even when they are really hard for me. |
| **Conscientiousness** | + | When I finish my homework, I check it many times to see if I did it correctly. |
|  | + | I play only when I finished my homework. |
|  | + | I keep my bedroom tidy. |
| **Growth mindset** | + | You can always become more clever. |
|  | - | You have a certain amount of talent, and you can't do much to change it. |
| **School liking** | + | Do you like to come to school? |
|  | - | Do you wish you could stay home from school? |
| **Class distraction** | + | I get easily distracted during class-time. |

# Analyses

### Pre-processing

Our tablet application produced text files for each task, and the data in these were extracted using a custom pipeline developed in Python (Dalmaijer, 2017; Van Rossum & Drake, 2011). This pipeline extracted trial-level data, computed the outcome measures outlined above, and standardised data within each measure. Two datasets with more than 20% missing values (38 and 42%) were excluded from analysis. After standardisation, missing values for included datasets were imputed using 9-nearest neighbours models fitted on 397 complete datasets. In total, 55 datasets had 1 value imputed, 5 had 2, 4 had 3, 40 had 4, 13 had 5, 1 had 6, 2 had 8, and 2 had 9.

### Principal component and parallel analyses

We employed factor analysis with orthogonal (varimax) rotation on 19 developmental measures to characterise the dimensions along which the children in our sample vary. Rotated components (RCs) with eigenvalues over 1 are reported, a crude method for identifying those RCs which explain more variance than any single variable (Kaiser’s criterion). Further scrutinisation occurred in a parallel analysis; a more conservative approach of extracting components. In a parallel analysis, eigenvalues of pre-rotated components were compared to a reference distribution. This was made up of eigenvalues that were computed in 1000 iterations, in each of which the original values were permuted within each value. This ensured equal distributions between the original and permuted data, but no systematic correlations between them. Only those RCs with eigenvalues greater than the respective component derived from the parallel analysis were deemed significant (Horn, 1965).

### Clustering

Cluster analysis aims to characterise individuals as members of discrete groups, and examples of its application include identifying distinct phenotypes of search organisation in adults (Benjamins et al., 2019) and among struggling learners (Bathelt et al., 2018). Here, we take a similar approach.

Cluster analyses suffer from the “curse of dimensionality” (Bellman, 1957): The more features a dataset has, the less likely it is to reliably identify clusters. To avoid this, we reduced the dimensionality of our data from 19 features to 2, using multi-dimensional scaling (Kruskal, 1964a, 1964b): this projects high-dimensional data into 2 dimensions while retaining relative inter-sample distances.

After dimensionality reduction, we applied k-means clustering (for a historical overview of the method, see (Jain, 2010)). The k-means algorithm requires a user to choose a value for k, the number of clusters, and then randomly generates k centroids. Samples are assigned to the centroid that is closest to them, after which a new centroid is computed as the average of all assigned samples. This continues over several iterations, until the centroid locations are stable. At this point, each sample is considered to be part of the cluster to which centroid it is closest.

The quality of a cluster solution can be expressed in terms of how representative each cluster centroid is of its assigned samples. For each sample, a silhouette coefficient can be computed from the ratio between the distances between a sample, its assigned centroid, and the centroid of the closest cluster that it was not assigned to (Rousseeuw, 1987). A silhouette coefficient of 1 means that a sample is perfectly aligned with its assigned cluster centroid, a value of 0 that it is as far from its assigned centroid as it is from the closest other centroid, and a value of -1 means that a sample is perfectly aligned with a the centroid of a cluster that it was not assigned to. The silhouette coefficients of all samples can be averaged together to compute a marker of overall quality (sometimes labelled “cluster coefficient”), with values over 0.5 being considered evidence for the presence of clusters within a dataset (Kaufman & Rousseeuw, 1990), while values below suggest no clear sub-groups are present.

To find the number of clusters that best described the current dataset, we ran k-means analyses with k=2 to k=10 (Figure S1 shows the solutions for k=1 through k=8). The best fitting solution was no subgroups, because none of the k-means solutions produced an average silhouette coefficient over 0.5 (this should have appeared as a peak in the bottom-right panel in Figure S1). This was also visually evident from the clear lack of clusters in the scatterplots.


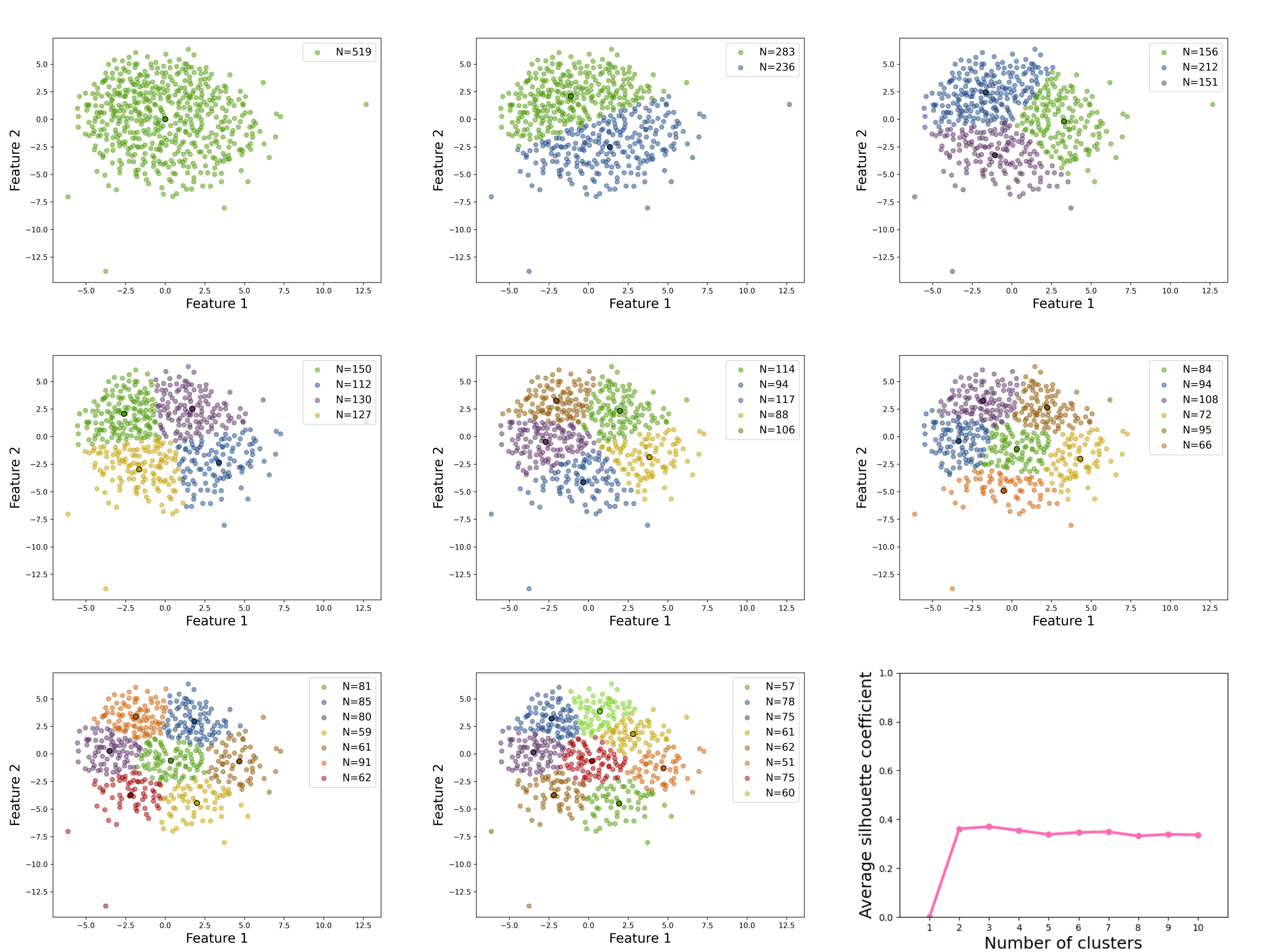


**Supplementary Figure S1** – The first eight panels show the sample (plotted features 1 and 2 are the result of multi-dimensional scaling on all variables listed in Table 2), with colours delineating subgroups indicated by a k-means procedure with k=2 through k=8. The bottom-right panel shows the silhouette score for each solution, illustrating that no value is above the 0.5 threshold for “clustering”, and no peak is evident.

### Network analysis

While clustering approaches focus on characterising sub-groups of individuals, we employed the following analysis to investigate the relationship between factors. Here, we cast measured factors such as “spatial short-term memory” (dot matrix performance), “depression” (RCADS depression symptoms sub-score), and “deprivation” (postcode-based deprivation index) as nodes in a network. Within a network, factors can be connected to each other via edges. The advantage of this approach is that it can clarify *how* factors relate to each other. Psychological network analysis is a relatively novel method, and has been employed in characterising symptoms within psychopathology (Borsboom & Cramer, 2013; Fried et al., 2017), psychological factors (Schmittmann et al., 2013), and resilience factors to mental health problems (Fritz et al., 2019).

For example, factors A, B, and C could show a high correlation with each other, but a network analysis can show that factor A does not directly relate to factor C, but that both A and C are related to B. In other words, network analysis shows direct relations and mediations between variables.

To visualise the network, we employed multi-dimensional scaling on the transposed dataset. This is as described above (under “Clustering”), although now we consider each factor as an observation, and each participant as a different feature. In the resulting solution, factors that are closer together showed more similar response patterns across participants.

We computed edge weights through partial correlation, which quantifies the uniquely explained variance of one factor on another. For example, to compute the partial correlation between factors A and B among factors A-Z, one would first compute a linear regression of B with C-Z as predictors, and then compute the correlation between A and the residuals of B. Partial correlation represents the unique relation between A and B after accounting for all other measured factors.

To distinguish between meaningful and spurious partial correlations, we employed a more liberal and a more conservative approach. The liberal approach constituted LASSO regularisation (Tibshirani, 1996, 2011), using a coordinate descent algorithm (Friedman et al., 2010; Kim et al., 2007) implemented in scikit-learn (Pedregosa et al., 2011), which sets spurious correlations to 0. What is considered a spurious correlation depends on a tuning parameter that we set through 5-fold cross validation.

A more conservative approach to limit spurious partial correlations was through bootstrapping (Efron, 1979) to estimate the probability of each edge occurring, and the variability of each edge’s strength. In each of 3000 iterations, we sampled with replacement from all observations, and computed a partial correlations in the resampled data. We could thus calculate in how many iterations each possible edge appeared, i.e. had a partial correlation with a p value below 0.05. We compared this against the expected number of edges to occur through chance, defined as the 95^th^ percentile of the number of edges observed in all iterations, or the Bonferroni-corrected 99.97^th^ percentile. We considered edges that appeared in more iterations than chance to be “statistically significant”. The bootstrapped 95% confidence intervals of edge weights were defined as the 2.5^th^ and 97.5^th^ percentile of all estimated partial correlations (Epskamp et al., 2018).

A more qualitative approach to interpreting psychological networks is by incrementally increasing the LASSO tuning parameter λ until no connections remain (at the highest absolute correlation, at 0.54 in this dataset), and estimating the surviving edges at each step (Epskamp & Fried, 2018). This provides insight into which connections are the most reliable (Supplementary Figure S2).

**
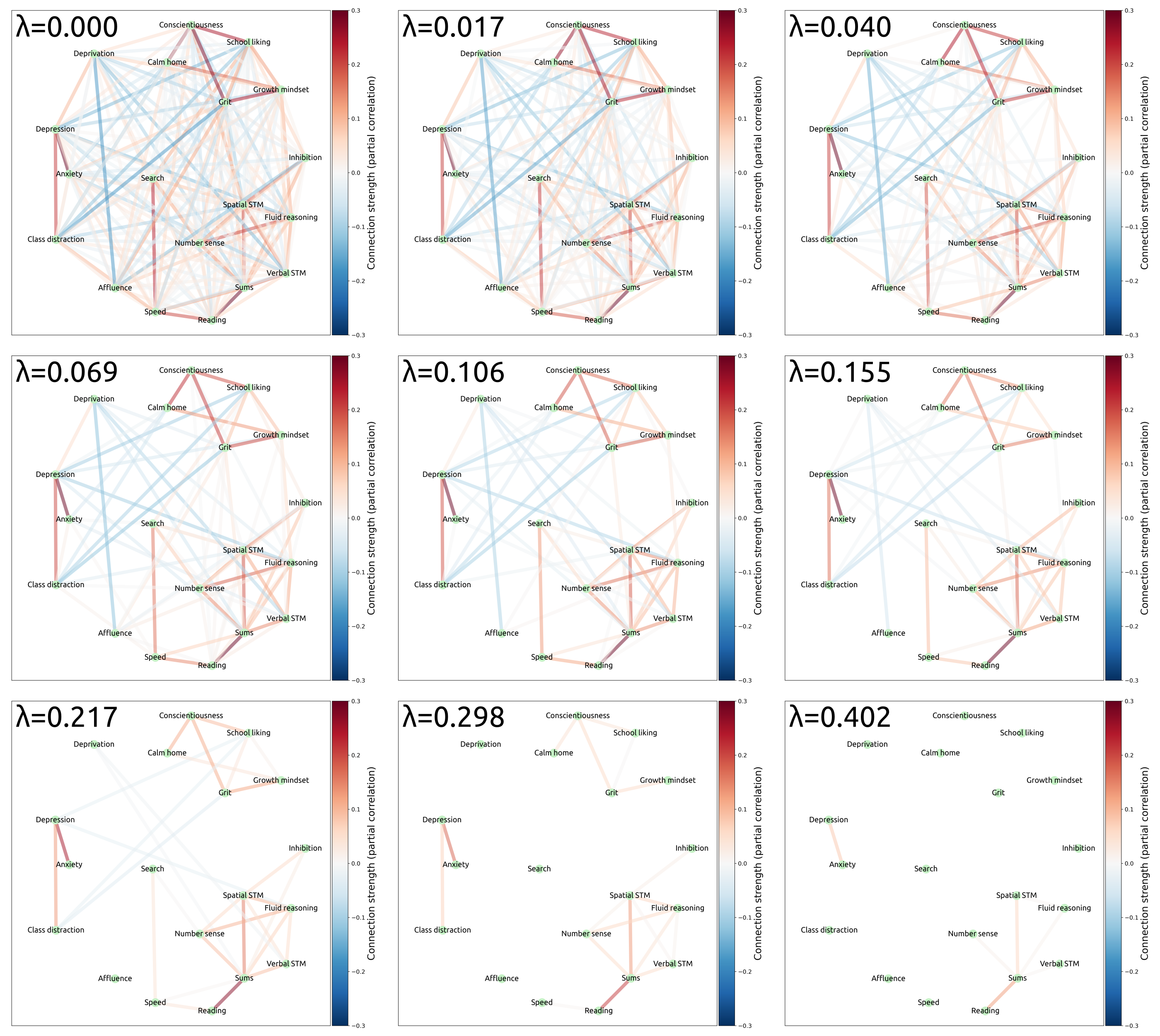
**

**Supplementary Figure S2** – Each panel shows the result of a network analysis through partial correlation with LASSO regularisation. The tuning parameter λ determines how strongly connections are penalised. As it increases towards the maximum absolute zero-order correlation, fewer networks survive, until finally none remain. The λ parameter thus governs the continuum between discovery and caution.

# Supplementary References

Barbaranelli, C., Caprara, G. V., Rabasca, A., & Pastorelli, C. (2003). A questionnaire for measuring the Big Five in late childhood. *Personality and Individual Differences*, *34*(4), 645–664. https://doi.org/10.1016/S0191-8869(02)00051-X

Bathelt, J., Holmes, J., Astle, D. E., & The CALM Team. (2018). Data-Driven Subtyping of Executive Function–Related Behavioral Problems in Children. *Journal of the American Academy of Child & Adolescent Psychiatry*, *57*(4), 252-262.e4. https://doi.org/10.1016/j.jaac.2018.01.014

Bellman, R. (1957). *Dynamic programming*. Princeton University Press.

Benjamins, J. S., Dalmaijer, E. S., Ten Brink, A. F., Nijboer, T. C. W., & Van der Stigchel, S. (2019). Multi-target visual search organisation across the lifespan: cancellation task performance in a large and demographically stratified sample of healthy adults. *Aging, Neuropsychology, and Cognition*, *26*(5), 731–748. https://doi.org/10.1080/13825585.2018.1521508

Bishop, D. V. M. (2018, September 15). An index of neighbourhood advantage from English postcode data. *BishopBlog*. http://deevybee.blogspot.com/2018/09/an-index-of-neighbourhood-advantage.html

Borsboom, D., & Cramer, A. O. J. (2013). Network Analysis: An Integrative Approach to the Structure of Psychopathology. *Annual Review of Clinical Psychology*, *9*(1), 91–121. https://doi.org/10.1146/annurev-clinpsy-050212-185608

Cattell, R. B. (1940). A culture free intelligence test. *Journal of Educational Psychology*, *31*, 161–180.

Dalmaijer, E. S. (2017). *Python for experimental psychologists*. Routledge.

Dalmaijer, E. S., Li, K. M. S., Gorgoraptis, N., Leff, A. P., Cohen, D. L., Parton, A., Husain, M., & Malhotra, P. A. (2018). Randomised, double-blind, placebo-controlled crossover study of single-dose guanfacine in unilateral neglect following stroke. *Journal of Neurology, Neurosurgery & Psychiatry*, jnnp-2017-317338. https://doi.org/10.1136/jnnp-2017-317338

Dalmaijer, E. S., Van der Stigchel, S., Nijboer, T. C. W., Cornelissen, T. H. W., & Husain, M. (2015). CancellationTools: All-in-one software for administration and analysis of cancellation tasks. *Behavior Research Methods*, *47*(4), 1065–1075. https://doi.org/10.3758/s13428-014-0522-7

Department for Communities and Local Government, U. K. (2015). *The English Index of Multiple Deprivation (IMD) 2015 - Guidance*. https://www.gov.uk/government/statistics/english-indices-of-deprivation-2015

Efron, B. (1979). Bootstrap Methods: Another Look at the Jackknife. *The Annals of Statistics*, *7*(1), 1–26. https://doi.org/10.1214/aos/1176344552

Epskamp, S., Borsboom, D., & Fried, E. I. (2018). Estimating psychological networks and their accuracy: A tutorial paper. *Behavior Research Methods*, *50*(1), 195–212. https://doi.org/10.3758/s13428-017-0862-1

Epskamp, S., & Fried, E. I. (2018). A tutorial on regularized partial correlation networks. *Psychological Methods*, *23*(4), 617–634. https://doi.org/10.1037/met0000167

Fried, E. I., van Borkulo, C. D., Cramer, A. O. J., Boschloo, L., Schoevers, R. A., & Borsboom, D. (2017). Mental disorders as networks of problems: a review of recent insights. *Social Psychiatry and Psychiatric Epidemiology*, *52*(1), 1–10. https://doi.org/10.1007/s00127-016-1319-z

Friedman, J., Hastie, T., & Tibshirani, R. (2010). Regularization paths for generalized linear models via coordinate descent. *Journal of Statistical Software*, *33*(1), 1–22.

Fritz, J., Stochl, J., Fried, E. I., Goodyer, I. M., van Borkulo, C. D., Wilkinson, P. O., & van Harmelen, A.-L. (2019). Unravelling the complex nature of resilience factors and their changes between early and later adolescence. *BMC Medicine*, *17*(1), 203. https://doi.org/10.1186/s12916-019-1430-6

Furlong, M. J., You, S., Renshaw, T. L., O’Malley, M. D., & Rebelez, J. (2013). Preliminary Development of the Positive Experiences at School Scale for Elementary School Children. *Child Indicators Research*, *6*(4), 753–775. https://doi.org/10.1007/s12187-013-9193-7

Gebuis, T., & Reynvoet, B. (2011). Generating nonsymbolic number stimuli. *Behavior Research Methods*, *43*(4), 981–986. https://doi.org/10.3758/s13428-011-0097-5

Jain, A. K. (2010). Data clustering: 50 years beyond K-means. *Pattern Recognition Letters*, *31*(8), 651–666. https://doi.org/10.1016/j.patrec.2009.09.011

Kaufman, L., & Rousseeuw, P. J. (Eds.). (1990). *Finding Groups in Data*. John Wiley & Sons, Inc. https://doi.org/10.1002/9780470316801

Kim, S.-J., Koh, K., Boyd, S., & Gorinevsky, D. (2007). A interior-point method for large-scale L1-regularized least squares. *IEEE Journal of Selected Topics in Signal Processing*, *1*(4), 606–617.

Kruskal, J. (1964a). Multidimensional scaling by optimizing goodness of fit to a nonmetric hypothesis. *Psychometrika*, *29*(1), 1–27.

Kruskal, J. (1964b). Nonmetric multidimensional scaling: A numerical method. *Psychometrika*, *29*(2), 115–129. https://doi.org/10.1007/BF02289694

Malhotra, P. A., Parton, A. D., Greenwood, R., & Husain, M. (2006). Noradrenergic modulation of space exploration in visual neglect. *Annals of Neurology*, *59*(1), 186–190. https://doi.org/10.1002/ana.20701

Muris, P., Meesters, C., & Schouten, E. (2002). A brief questionnaire of DSM-IV-defined anxiety and depression symptoms among children. *Clinical Psychology & Psychotherapy*, *9*(6), 430–442. https://doi.org/10.1002/cpp.347

Odic, D., & Starr, A. (2018). An Introduction to the Approximate Number System. *Child Development Perspectives*, *12*(4), 223–229. https://doi.org/10.1111/cdep.12288

Parton, A. D., Malhotra, P. A., Nachev, P., Ames, D., Ball, J., Chataway, J., & Husain, M. (2006). Space re-exploration in hemispatial neglect. *NeuroReport*, *17*(8), 833–836.

Pedregosa, F., Varoquaux, G., Gramfort, A., Michel, V., Thirion, B., Grisel, O., Blondel, M., Prettenhofer, P., Weiss, R., Dubourg, V., Vanderplas, J., Passos, A., Cournapeau, D., Brucher, M., Perrot, M., & Duchesnay, E. (2011). Scikit-learn: Machine learning in Python. *Journal of Machine Learning Research*, *12*, 2825–2830.

Petrill, S. A., Pike, A., Price, T., & Plomin, R. (2004). Chaos in the home and socioeconomic status are associated with cognitive development in early childhood: Environmental mediators identified in a genetic design. *Intelligence*, *32*(5), 445–460. https://doi.org/10.1016/j.intell.2004.06.010

Rousseeuw, P. (1987). Silhouettes: A graphical aid to the interpretation and validation of cluster analysis. *Journal of Computational and Applied Mathematics*, *20*, 53–65. https://doi.org/10.1016/0377-0427(87)90125-7

Schmittmann, V. D., Cramer, A. O. J., Waldorp, L. J., Epskamp, S., Kievit, R. A., & Borsboom, D. (2013). Deconstructing the construct: A network perspective on psychological phenomena. *New Ideas in Psychology*, *31*(1), 43–53. https://doi.org/10.1016/j.newideapsych.2011.02.007

Tibshirani, R. (1996). Regression shrinkage and selection via the lasso. *Journal of the Royal Statistical Society: Series B (Methodology)*, *58*(1), 267–288.

Tibshirani, R. (2011). Regression shrinkage and selection via the lasso: a retrospective: Regression Shrinkage and Selection via the Lasso. *Journal of the Royal Statistical Society: Series B (Statistical Methodology)*, *73*(3), 273–282. https://doi.org/10.1111/j.1467-9868.2011.00771.x

Torsheim, T., Levin, K. A., Schnohr, C., Mazur, J., Niclasen, B., Currie, C., & the FAS Development Study Group. (2016). Psychometric Validation of the Revised Family Affluence Scale: a Latent Variable Approach. *Child Indicators Research*, *9*(3), 771–784. https://doi.org/10.1007/s12187-015-9339-x

Van Rossum, G., & Drake, F. L. (2011). *Python Language reference manual*. Network Theory Ltd.
